# Supplementary material for: The Evolution, Genomic Epidemiology, and Transmission Dynamics of Tembusu Virus
Source: Viruses. 2022 Jun 7;14(6):1236. doi: 10.3390/v14061236 (PMC9227414; doi:10.3390/v14061236)
Supplement: Supplementary file 1 [file viruses-14-01236-s001.zip › viruses-1688358 Supplement Table S2.pdf]

Supplementary Table S2 Bayes Factor (BF) and Posterior Probability (PP) tests for TMUV transmission links in Asia.

From	To	Bayes Factory (BF>15)	Posterior Probability (PP>0.5)
CNGD	CNGX	799.1952499	0.981222454
CNBJ	CNGD	289.2384153	0.949778398
CNJS	CNHN	78.22429416	0.836458809
CNFJ	CNSC	63.31736892	0.805446846
CNSD	CNJS	34.02859108	0.689917408
CNFJ	TH	28.89645428	0.653905507
CNGD	CNSX	27.32305184	0.641127887
CNSD	CNZJ	26.6222587	0.635127961
CNSD	CNFJ	23.74286097	0.608214713
MY	CNSD	23.03628211	0.60099258
CNSD	CNAH	19.45298753	0.55984494
CNJS	CNJX	19.12284424	0.55562277
CNHB	CNAH	18.43872231	0.546610536
CNSD	CNHB	17.37136831	0.531795904
CNHB	CNBJ	16.06412072	0.512277626
CNSD	CNHLJ	15.73424902	0.507092505
