# Supplementary material for: The Evolution, Genomic Epidemiology, and Transmission Dynamics of Tembusu Virus
Source: Viruses. 2022 Jun 7;14(6):1236. doi: 10.3390/v14061236 (PMC9227414; doi:10.3390/v14061236)
Supplement: Supplementary file 1 [file viruses-14-01236-s001.zip › viruses-1688358-supplementary Table S1.pdf]

Supplementary Table S1 Referenced TMUV strains used in phylogenetic analysis

No.	GenBank Accession No.	Data (Year/Month/Day)	Region
1	KJ740748	2013.11.06	CNAH
2	KJ958533	2012.05.12	CNAH
3	KM102539	2010.03.26	CNAH
4	MN649267	2014.09.26	CNAH
5	MT708901	2019.10.15	CNAH
6	JF270480	2010.06.16	CNBJ
7	JQ920420	2012.04.10	CNBJ
8	JQ920421	2012.04.10	CNBJ
9	JQ920423	2012.04.10	CNBJ
10	JQ920424	2012.04.10	CNBJ
11	JQ920425	2012.04.10	CNBJ
12	JQ920426	2013.08.23	CNBJ
13	KT876991	2015.10.05	CNBJ
14	MK542820	2019.02.18	CNBJ
15	MN649265	2013.08.21	CNBJ
16	MT108702	2019.04.04	CNBJ
17	JQ928189	2010.08.23	CNFJ
18	JX196334	2010.07.10	CNFJ
19	KX977551	2011.02.25	CNFJ
20	KX977553	2011.05.03	CNFJ
21	KX977554	2011.11.09	CNFJ
22	KX977555	2010.12.07	CNFJ
23	JX549382	2012.01.15	CNGD
24	KT159713	2014.03.03	CNGD
25	KT824876	2015.03.15	CNGD
26	KX686570	2015.07.01	CNGD
27	KX686571	2015.09.15	CNGD
28	KX686572	2015.03.11	CNGD
29	KX686573	2014.12.08	CNGD
30	KX686574	2015.04.23	CNGD
31	KX686575	2013.11.02	CNGD
32	KX686576	2013.07.08	CNGD
33	KX686577	2013.10.18	CNGD
34	KX686578	2011.06.07	CNGD
35	KX686579	2015.09.09	CNGD
36	KX686580	2014.11.06	CNGD
37	KX977550	2011.09.21	CNGD
38	MT447092	2018.10.15	CNGD
39	KC990542	2011.02.15	CNGX
40	KJ700462	2013.05.10	CNGX
41	KM275940	2013.01.22	CNGX

42	KM275941	2013.06.20	CNGX
43	KP861859	2013.07.25	CNGX
44	MK889501	2019.02.18	CNGX
45	MN649263	2015.04.26	CNGX
46	MN649264	2012.09.28	CNGX
47	JQ595407	2010.09.15	CNHB
48	KJ489355	2012.12.03	CNHB
49	KX452096	2014.01.13	CNHB
50	MK907880	2018.11.17	CNHB
51	MN649262	2010.10.30	CNHB
52	MN649266	2016.07.13	CNHB
53	MN966679	2019.09.08	CNHB
54	MN966680	2019.09.23	CNHB
55	JQ289550	2010.10.01	CNHLJ
56	KC136210	2011.01.20	CNHLJ
57	KF192951	2010.04.25	CNHLJ
58	KJ782377	2012.06.30	CNHLJ
59	KJ782378	2012.06.30	CNHLJ
60	KJ782379	2012.06.30	CNHLJ
61	KJ782380	2012.06.30	CNHLJ
62	MN649261	2015.03.26	CNHN
63	AB917090	2012.12.01	CNJS
64	JF895923	2010.09.15	CNJS
65	JQ920422	2012.04.10	CNJS
66	JX273153	2010.08.10	CNJS
67	KM188953	2012.11.05	CNJS
68	KR869106	2014.08.20	CNJS
69	KY626659	2015.09.08	CNJS
70	KY810818	2015.09.08	CNJS
71	KY810819	2015.09.08	CNJS
72	KP096415	2014.05.15	CNJX
73	KM233707	2013.11.25	CNSC
74	MW143073	2019.08.30	CNSC
75	JX965381	2010.06.30	CNSD
76	KC333867	2012.06.12	CNSD
77	KC990540	2010.10.15	CNSD
78	KC990541	2012.01.15	CNSD
79	KC990543	2010.11.15	CNSD
80	KC990544	2010.12.15	CNSD
81	KC990545	2012.03.15	CNSD
82	KF557893	2012.10.26	CNSD
83	KF557894	2013.10.20	CNSD
84	KF826767	2012.12.15	CNSD

85	KJ740745	2013.10.01	CNSD
86	KJ740746	2011.11.01	CNSD
87	KJ740747	2013.11.01	CNSD
88	MH748542	2014.06.30	CNSD
89	MN649260	2010.12.14	CNSD
90	MT951412	2020.01.19	CNSD
91	MT951413	2019.12.20	CNSD
92	KP742476	2015.02.04	CNSH
93	MH414568	2010.06.30	CNSH
94	KM066945	2013.11.22	CNSX
95	MN747003	2019.06.20	CNTW
96	MW821486	2020.12.15	CNTW
97	MW922032	2019.10.23	CNTW
98	AB917088	2010.08.01	CNZJ
99	AB917089	2012.12.25	CNZJ
100	JF459991	2010.05.25	CNZJ
101	JQ314464	2010.06.20	CNZJ
102	JQ314465	2010.12.08	CNZJ
103	KX977552	2010.11.29	CNZJ
104	JX477685	2013.11.22	MY
105	KX097989	2012.06.30	MY
106	KX097990	2012.06.30	MY
107	MH414569	2018.05.30	MY
108	KF573582	2013.08.23	TH
109	KR061333	2013.11.15	TH
110	MH460536	2015.11.15	TH

CNAH: Anhui Province, China; CNBJ: Beijing City, China; CNFJ: Fujian Province, China; CNGD: Guangdong Province, China; CNGX: Guangxi Province, China; CNHB: Hubei, Province; CNHLJ: Heilongjiang Province, China; CNJS: Jiangsu Province, China; CNJX: Jiangxi Province, China; CNSC: Sichuan Province, China; CNSD: Shandong Province, China; CNSH: Shanghai City, China; CNSX: Shanxi Province, China; CNTW: Taiwan Province, China; CNZJ: Zhejiang Province, China; MY: Malaysia; TH: Thailand.
